# On the antimicrobial properties and endurance of eugenol and 2-phenylphenol functionalized sol-gel coatings

**DOI:** 10.1016/j.heliyon.2024.e29146

**Published:** 2024-04-03

**Authors:** Ana Suárez-Vega, Gemma Berriozabal, Juan Perez de Iriarte, Jaione Lorenzo, Noelia Álvarez, Santiago Dominguez-Meister, Sara Insausti, Edurne Rujas, Jose L. Nieva, Marta Brizuela, Iñigo Braceras

**Affiliations:** a TECNALIA, Basque Research and Technology Alliance (BRTA), Mikeletegi Pasealekua 2, Donostia-San Sebastián, Spain; b Instituto Biofisika (CSIC-UPV/EHU), University of the Basque Country (UPV/EHU), PO Box 644, 48080, Bilbao, Spain; c Department of Biochemistry and Molecular Biology, University of the Basque Country (UPV/EHU), PO Box 644, 48080, Bilbao, Spain; d Ikerbasque, Basque Foundation for Science, 48013, Bilbao, Spain; e Pharmacokinetic, Nanotechnology and Gene Therapy Group, Faculty of Pharmacy, University of the Basque Country UPV/EHU, 01006, Vitoria-Gasteiz, Spain; f Bioaraba, Microbiology, Infectious Disease, Antimicrobial Agents, and Gene Therapy, 01006, Vitoria-Gasteiz, Spain

**Keywords:** Eugenol, 2-Phenylphenol, Sol-gel, Antibacterial, Virucide, Durability

## Abstract

Preventing microbiological surface contamination in public spaces is nowadays of high priority. The proliferation of a microbial infection may arise through air, water, or direct contact with infected surfaces. Chemical sanitization is one of the most effective approaches to avoid the proliferation of microorganisms. However, extended contact with chemicals for cleaning purposes such as chlorine, hydrogen peroxide or ethanol may lead to long-term diseases as well as drowsiness or respiratory issues, not to mention environmental issues associated to their use. As a potentially safer alternative, in the present work, the efficacy and endurance of the antimicrobial activity of different sol-gel coatings were studied, where one or two biocides were added to the coating matrix resulting on active groups exposed on the surface. Specifically, the coating formulations were synthesized by the sol-gel method. Using the alkoxide route with acid catalysis a hybrid silica-titania-methacrylate matrix was obtained where aromatic liquid eugenol was added with a double function: as a complexing agent for the chelation of the reaction precursor titanium isopropoxide, and as a biocide. In addition, 2-Phenylphenol, ECHA approved biocide, has also been incorporated to the coating matrix. The antibacterial effect of these coatings was confirmed on Gram-positive (*Staphylococcus aureus*) and Gram-negative bacteria (*Escherichia coli*). Additionally, the coatings were non cyto-toxic and displayed virucidal activity. The coating chemical composition was characterized by ^29^Si NMR, and ATR-FTIR. Furthermore, the thickness and the mechanical properties were characterized by profilometry and nanoindentation, respectively. Finally, the durability of the coatings was studied with tribology tests. Overall, our data support the efficacy of the tested sol-gel coatings and suggest that added features may be required to improve endurance of the antimicrobial effects on operational conditions**.**

## Highlights

• Eugenol and 2-phenylphenol have been successfully incorporated into sol-gel coatings.
• Some coatings show antibacterial activity against both Gram-positive and negative bacteria.
• The coatings were also non cyto-toxic and displayed virucidal activity.
• Antimicrobial activity endurance against cleaning processes is however limited.

## 1 Introduction

The fast spread of microorganisms may cause particularly harmful situations. Different modes of transmission exist for viral or bacterial infections, including the spread through air and/or water, as well as contact with contaminated surfaces. The risk of transmission from a brief contact with a single contaminated surface is generally low, however, as individuals are in contact with multiple contaminated surfaces, the population density is high, the duration of a stay in an existing location increases, and the effectiveness of mitigation measures is poor (e.g. surface cleansing and handwashing), the risk of infection's transmission escalates [1]. Pathogen transmission is not limited to healthcare settings like hospitals or medical centres. It can also occur in community spaces such as public transportation, where numerous individuals come into contact with various surfaces such as seats, doorknobs, railings, tables, and armrests [1,2].

In this context, nowadays, there is a high interest in avoiding pathogenic surface contamination. One possible strategy to address this issue is the development of antimicrobial coatings capable of displaying both antibacterial and virucidal activity.

Antimicrobial coatings can typically hinder initial bacterial adhesion [3,4], show contact-killing ability [5] or release biocide agents [[6], [7], [8], [9], [10], [11], [12]]. Contact-killing coatings involve the use of surface-attached biocidal agents, which can range from natural biomacromolecules to synthetic compounds or polymers. On the other hand, biocide-release coatings typically rely on the controlled release of antibiotics [6,7], metal nanoparticles [[8], [9], [10]] or other biocides that are preloaded or embedded within the coating [11,12]. However, a major drawback of antimicrobial metallic derivatives is their toxicity-inducing potential [11].

Among the coating deposition technologies, sol-gel synthesized coatings have emerged as highly promising solutions. This synthesis process offers several advantages, such as the ability to produce exceptionally pure materials at low synthesis temperatures and without requiring a washing purifying step, which minimizes the environmental impact during the coating synthesis [13]. Furthermore, the sol-gel process is highly suitable: it enables the synthesis of hybrid organic-inorganic materials that can easily incorporate functional groups, e.g. antimicrobial. Additionally, these coatings can be applied to a wide range of substrates using cost-effective simple and viable techniques like dip-coating [14].

The alkoxide route is one of the most employed synthesis method. It is a wet chemical method, which involves the hydrolysis and condensation of chemical precursors, typically metal alkoxides, in the presence of an acidic or basic catalyst within an alcohol-based solution. This results in the formation of a colloidal suspension called a “sol”, which is then deposited onto the desired substrate using techniques such as dip-coating (used in this work) or spraying. Subsequent solvent evaporation leads to the formation of a fluid phase trapped within a non-fluid network known as a “gel”. To complete the process, a final thermal treatment step is usually required to eliminate any unreacted compounds, as well as physiosorbed water and solvent, while promoting the condensation and polymerization of the gel to get a dense and highly crosslinked network [15].

Silicon alkoxides are the most commonly used precursors in sol-gel reactions. Alkoxides of other transition metals such as zirconium, titanium or aluminium can also be of interest. The main difference is their reactivity: zirconium or titanium are more electropositive than silicon and therefore react heavily with water. Thus, they need to be sterically hindered to control the hydrolysis and condensation reactions. In contrast, silicon alkoxides, especially those with large functional groups or longer alkoxide chains, often require the addition of a catalyst to facilitate the hydrolysis and condensation reactions [16].

These precursors can be either completely inorganic, or they can possess an organic moiety. The resulting inorganic coatings exhibit improved durability, scratch, thermal and chemical resistance, as well as better adhesion to the substrate. The addition of organic functional groups enhances coating density, flexibility, mechanical properties, and the ability to form thicker coatings [14,16,17].

The inclusion of two organic phenolic compounds: eugenol and 2-phenylphenol (OPP) with antimicrobial activity [18] is proposed and explored in this work. Eugenol is a naturally occurring phenolic monoterpenoid that falls under the class of phenylpropanoids. It is commonly found in various aromatic herbal plants including clove, tulsi, cinnamon, nutmeg, and pepper. It is primarily isolated from the clove plant (*Eugenia caryophyllata*) [19] and it exhibits pharmacological activity against various bacteria, including both Gram-negative and Gram-positive strains [20,21]. Eugenol alters the membrane, affects the transport of ions and ATP and changes the fatty acid profile of different bacteria, and acts against different bacterial enzymes, including ATPase, histidine carboxylase, amylase and protease [22,23]. The antimicrobial effects are significantly influenced by the side chain structure and functional groups' nature present in phenolic compounds [24]. The bioactivity of eugenol is attributed to the presence of aromatic rings in its chemical structure, which contain hydroxyl (-OH) and methoxy (-OCH_3_) functional groups [25,26]. The second phenolic compound used in this study is 2-phenylphenol (OPP), a widely used biocidal agent in multiple industrial applications. Its fungicidal and bactericidal properties make it valuable for preserving food, wood, and textiles. Additionally, it is used in personal care products, agriculture, and as a disinfectant [27].

Although the incorporation of eugenol in different hybrid matrix had been previously studied [[28], [29], [30], [31], [32], [33]], to the best of the authors knowledge, neither its combination as a biocide and a chelating agent nor the combination of eugenol and OPP into sol-gel coatings had not been previously explored. In addition to the intrinsic coating properties, the antimicrobial and non-toxic properties of the coatings have been evaluated with positive results, against both bacteria, Gram-positive (*Staphylococcus aureus*) and Gram-negative (*Escherichia coli*), and viruses. Furthermore, the endurance of this antimicrobial activity after cycles simulating operative conditions has also been assessed.

## 2 Material and methods

### 2.1 Materials

The solvent n-propanol (>99%) was acquired from Scharlab, S.L. (Sentmenat, Spain). The inorganic precursors, silicon, and titanium alkoxides: tetraethyl orthosilicate (TEOS, 98%) and titanium isopropoxide (TISP, 97%) respectively, were purchased from Acros Organics (Geel, Belgium). Hybrid organic-inorganic precursor methacrylate functional silane, Dynasylan® MEMO, (MAPTS, ≤100%) was purchased from Evonik Industries AG (Essen, Germany). Organic precursors Eugenol (natural, ≥98 %, FG) and 2-Phenylphenol (OPP, 99 %) were acquired from MERCK (Sigma-Aldrich, St. Louis, MO, USA). All these precursors were used as received. Sulfuric acid (H_2_SO_4_, 95–97%), and nitric acid (HNO_3_, 67%) were purchased from Scharlab S.L. (Sentmenat, Spain) and used to prepare a 0.1 M and 0.01 M solution respectively, to act as a catalyst in the sol synthesis. Nitric acid (HNO_3_, 67%) and hydrofluoric acid (HF, 40 % solution) were acquired from Scharlab S.L. (Sentmenat, Spain) and were used to make a 10 % HNO_3_ and 1.5 % HF in deionized water solution for the pretreatment of the substrates. Trisodium phosphate and sodium dodecyl sulfate (SDS) were both bought from MERCK (Sigma-Aldrich, St. Louis, MO, USA).

Three types of substrates were used for the study of the biocide activity and other properties of the coatings. AISI 304L 50 × 50 mm test samples cut from a 1.4 mm sheet with a 2B finishing (cold rolled, annealed, pickled and wet skin-passed material; Acerinox, Spain) and the following chemical composition (%wt): C ≤ 0.030, Si ≤ 0.75, Mn ≤ 2.00, P ≤ 0.040, S ≤ 0.015, Cr 18.00–19.00, Ni 8.00–10.00 were used for the experiments performed with Gram-negative bacteria (*Escherichia coli*). In addition, glass coverslips of 76 × 50 mm and a thickness of 1 mm (reference BPB038) were purchased from AUXILAB (Navarra, Spain) and used for the experiments carried out with the Gram-positive bacteria (*Staphylococcus aureus*) as well as for the durability essays. Finally, for the virucidal experiments glass coverslips of 24 × 24 mm and a thickness of 0.13–0.16 mm (reference 23124) were obtained from LABOLAN (Navarra, Spain).

### 2.2 Synthesis of the coatings

Four different coatings were studied (Table 1, Table 2). The first one is silicon-methacrylate functional based and it was prepared with a silicon alkoxide and an organic functional silicon-alkoxide (control/reference coating); and the other three are silicon-titanium- methacrylate functional based and were prepared with the previous silicon and organic functional silicon alkoxide, as well as a complexed titanium alkoxide. All coatings were synthesized following the alkoxide route of the sol-gel method [16] based on the formation of two stable and homogenous parts.

**Table 1:** Molar equivalence of the precursors for each of the liquid sol formulations (NOTE: Eu: eugenol).

Sol formulation	TEOS	MAPTS	HNO_3_	Eu	OPP	TISP	n-Propanol	H_2_SO_4_
**Si-MPT sol**	1	1	4					
**Si-MPT_OPP sol**	1	1	4		0.6			
**Si(eu)MPT sol**	1	1	4	0.5				
**Ti(eu) sol**				0.4		0.4	1	0.8

**Table 2:** Sol formulations (Table 1) used for each coating.

Coatings	Sol formulation
**Si-MPT**	Si-MPT sol
**SiTi(eu)-MPT**	Si-MPT sol + Ti(eu) sol
**SiTi(eu)-MPT_OPP**	Si-MPT_OPP sol + Ti(eu) sol
**SiTi(eu2)-MPT**	Si(eu)-MPT sol + Ti(eu) sol

For the first silicon-methacrylate functional coating (Si-MPT sol), a 1:1 molar equivalence mixture of TEOS and MAPTS was made and left stirring at room temperature (RT) for 5 min. Then, a 0.1 N HNO_3_ solution was added dropwise to the mixture (at a molar ratio of 4:1; HNO_3_ to TEOS) to accomplish the hydrolysis and condensation reactions. The complete mixture was allowed to age for 24 h at RT (Table 1, Si-MPT sol and Table 2, Si-MPT coating).

The second coating (silicon-titanium- methacrylate functional, SiTi(eu)-MPT) was synthesized by mixing two different parts: a first part was made as explained above (Table 1, Si-MPT sol). The second part was synthesized as follows: n-propanol was weighed, TISP was then added into the n-propanol followed by the slow addition of eugenol to chelate the titanium to reduce the high reactivity reaction kinetics that titanium alkoxides have in the presence of oxygen and/or water. The mixture was allowed to stir for 1 h at RT after which 0.1 M H_2_SO_4_ was added to catalyse the hydrolysis and condensation reactions of the titanium network. The complete titanium mixture was allowed to age for 24 h at RT (Table 1, Ti(eu) sol). After both parts were on stirring conditions for 24 h, they were mixed together (Table 2, SiTi(eu)-MPT).

The third coating (silicon-titanium-methacrylate functional, SiTi(eu)-MPT_OPP) was synthesized in the same way as SiTi(eu)-MPT. However, in this case, in the silicon-methacrylate functional sol, apart from TEOS, MAPTS, and HNO_3_ 0.01 M, OPP is also added to the formulation on a molar ratio 0.6:1 (OPP:TEOS) (Table 1, SiMPT_OPP sol) and then mixed with Ti(eu) sol (Table 2, SiTi(eu)-MPT_OPP).

Lastly, the fourth coating (silicon-titanium-methacrylate functional, SiTi(eu2)-MPT) was synthesized by mixing two different parts: a first part was made as explained above but also including eugenol on a molar ratio 0.5:1 (eugenol: TEOS) (Table 1, Si(eu)-MPT sol). The Si(eu)MPT sol was then mixed with Ti(eu) sol (Table 2, SiTi(eu2)-MPT) and complete mixture was allowed to age for 24 h at RT.

The synthesis molar ratios of the different silicon and titanium sol formulations are displayed in Table 1, Table 2.

### 2.3 Substrate preparation and coatings deposition

A pre-treatment was given to the different substrates to ensure a good adhesion of the coatings. Glasses were cleaned with ethanol and dried with oil-free compressed air. On the other hand, the stainless steel was treated with the following procedure: first, an alkaline cleaning was performed by immersing the steel coupons on a mixture composed of deionized water with 35 g/L trisodium phosphate and 0.5 g/L sodium dodecyl sulfate (SDS) for 90 s at 80 °C. An acid pickling bath was then prepared to activate the steel surface. The mixture consisted of 10 % HNO_3_ and 1.5 % HF in deionized water and panels were immersed on this solution for 15 min at RT. After the coupon's pre-treatment, samples were washed with deionized water, ethanol and dried with oil-free compressed air.

The coatings were deposited by the dip-coating technique. The method implies the immersion of the substrate in the solution and its withdrawal at a controlled homogeneous speed to ensure a specific coating thickness. Coating formulations were deposited on the different panels’ substrate by dip-coating using in house-made equipment with controlled immersion and withdrawal speeds. In this work, all coatings were withdrawn at 5 cm/min. After the coating deposition, the coatings were subjected to a thermal treatment of 120 °C for 1 h to ensure a good adhesion with the substrate through condensation reactions and to ensure the organic part was properly cured to achieve the needed crosslinking.

### 2.4 Characterization of the coatings

The viscosity was measured to obtain information of coating's formulations stability and thus, their pot-life. The viscosity was measured using the LOVIS 2000 M/ME rolling-ball viscometer, equipped with a 1.8 mm capillary, which allows measurements in the range of 2.5 and 30 mPa·s. The equipment calculates the liquid viscosity by measuring the rolling time of a ball through transparent and opaque liquids based on Hoeppler's falling ball principle. It measures the time a ball takes to move through the sample liquid.

The thickness of the coatings was measured on glass substrates using a Veeco Dektak 150 contact profilometer. The coating formulation was deposited onto the substrate as described above, at the withdrawal speeds of 5 cm/min. The coatings were then scratched after the thermal treatment. The coating thickness was determined by the measurement of the step height considering five scans across the 50-mm length scratch.

The roughness of the coatings deposited on stainless steel panels was also measured using the profilometer described above. Five 1.45-mm length scans were taken on each sample using a 2 μm radius stylus, 3 mg force and lengthwise resolution of 0.32 μm. The arithmetic average roughness, Ra, was calculated from the central 1.45 mm of the scan using a short and a long pass filter cut-off of 250 μm. The mean Ra of the five scans for each sample was then calculated.

The mechanical properties of the coatings such as hardness and scratch resistance were studied by the nano-indentation technique using a NHT3 Step 700 (Anton Paar).

The wettability of the coating surfaces was assessed in terms of water contact angles (Digidrop Contact Angle Meter, GBX Instruments). The contact angle was measured, at least fivefold, where the drop outer surface met the solid surface (Visiodrop software).

Liquid ^29^Si NMR assays were performed to define the environment of the silicon atoms of the coating formulation and to estimate the degree of hydrolysis and polycondensation of the final silicon–oxygen polymeric structure. All NMR experiments were measured at room temperature using a Bruker AVANCE III 500 MHz NMR at 11.7 T (for ^29^Si NMR 99.35 MHz). ^29^Si spectra were measured using Bruker's cpmg1d sequence using the following parameters: 16K acquisition points, relaxation time 5 s, 150 ppm bandwidth. Dummy scans 16 and 2048 averages. Data were zero filled to 64K and apodised using an exponential multiplication routine with 15 Hz of line broadening. The NMR tubes contain borosilicate, and, on some occasions, a broad signal was observed at around −110 ppm which could overlap with a signal from the sample. To avoid this, to all spectra, the spectrum of the tube was subtracted of the final one.

Attenuated Total Reflectance Fourier transform infrared (ATR-FTIR) spectroscopy was carried out on a FT-IR Nicolet™ iS™ 5 spectrometer. Spectra were acquired using a diamond attenuated total reflectance (ATR) crystal after 16 scans across the 4000–650 cm^− 1^ wavelength range.

### 2.5 Biological evaluation of the coatings

The antimicrobial activity of the surfaces was assessed *in vitro* both in terms of antibacterial and virucide activities.

In the case of the antibacterial activity of the coatings, it was assessed before and after subjecting the surfaces to tribology tests, which simulated cleaning procedures, to evaluate the durability of the antibacterial activity. The tribology tests were performed on a reciprocating mode, 30 mm long tracks, against 50 × 50 mm Scotch-Brite® sponge (3 M), in a MT/10/LF Tribometer (Microtest), for 7,200 cycles. AK-GERMA DT (Kliner Profesional S.A., Spain) cleaning product was applied in the coating sponge interface, which was subjected to a load of 10 N, and a lineal speed of 0.060 m/s.

The bactericide activity was tested against both *Escherichia coli* ATCC8739 (Gram-negative) and *Staphylococcus aureus* ATCC 6538P (Gram-positive) bacterial strains. The testing procedure followed was based on the ISO 22196 standard, on three replica per coating. The tests consisted basically of inoculating 0.4 ml of a bacterial concentration suspension (approximately 0.25–1 10^6^ cfu/ml) onto each sample surface, covering them with a non-toxic non-biocide polymer film, and incubating the samples for 24 h at 37 °C and 100 % of relative humidity. Afterwards the sample surfaces were extracted by placing each one in a sterile bag with 50 ml of sterile buffer in a stomacher (Masticator, Iul Instruments, Spain). The viable bacteria in each extract were counted (cfu: number of colony forming units), by pour plate method and the value of antimicrobial activity (R) was calculated, as the percentage of reductions of viable bacteria on the coated materials as cfu/cm^2^ with respect to the viable bacteria on the control surfaces.

Additionally, the potential cytotoxicity of the coatings was assessed following standard ISO 10993-5 by extraction method [34]. The aim of this test was to evaluate biologically the toxicity of the sample by lysis of the cells and inhibition of cell growth after putting the sample extract in contact during 24 h. The test sample and control samples were prepared following the ISO 10993-12 standard.

The antiviral activity of the coatings was assessed using pseudotyped *Vesicular stomatitis* G viruses (VSV-G) generated as previously described [35]. Briefly, plasmid pWPXL-GFP and pCMV8.91, encoding a green fluorescent protein (GFP) and an Env-deficient HIV-1 genome, respectively were co-transfected with a plasmid encoding the VSV-G Envelope (kindly provided by Dr. Patricia Villace, CSIC, Madrid). Transfection was performed in human embryonic kidney 293 (HEK293T) cells using the calcium phosphate method. The GFP-encoding plasmid bears LTR and Ψ sequences allowing insertion into the host cell genome and RNA encapsidation into virions, respectively. pCMV8.91, in contrast, lacks these sequences, and the virulence factors Vpu, Vpr, and Vif are mutated to avoid generation of an infectious progeny. 18 h after transfection, the medium was replaced with Optimem-Glutamax II (Invitrogen Ltd, Paisley, UK) without serum and the cells were further incubated for an additional 48 h. After that time, the PsVs were harvested, passed through 0.45 μm pore sterile filters (Millex® HV, Millipore NV, Brussels, Belgium) and stored in PBS at −80 °C. PsV Infectivity was determined using HeLa cells to stablish the amount needed to achieve a 10–15 % tissue culture infectious dose in the cell entry inhibition experiments.

The ability of the functional groups in the coatings to interfere with virus infectivity was determined by cell entry inhibition assays using VSV-G PsVs and HeLa cells. 5 drops of 5 μL each containing the VSV-G PsVs were distributed homogeneously through the surface of a glass cover previously treated with the coating agents. Each of the treated and untreated (control) samples were placed inside a well of a 6-well plate and incubated for 1 h at 37 °C to allow for PsV attachment. After incubation, unbound PsV particles were washed away with PBS. 11,000 HeLa cells were subsequently added per well in the presence of 30 μg/ml DEAE-dextran (Sigma-Aldrich, St-Louis, MO). Virucide activity levels were inferred after 72 h from the reduction in the number of GFP-positive cells as determined by flow cytometry using a BD FACS Calibur Flow Cytometer (Becton Dickinson Immunocytometry Systems, Mountain View, CA).

## 3 Results and discussion

### 3.1 Chemical characterization of formulations and coatings

The surroundings of the silicon atoms were qualitatively determined using liquid ^29^Si NMR analysis. This method provides an understanding of the level of hydrolysis and polycondensation that occurred on the network by following the evolution of the reaction of the different silicon alkoxides contained in the network. Two different silicon alkoxides were used in this work: TEOS and MAPTS. TEOS has four hydrolysable groups and therefore, five different signals are expected on the ^29^Si NMR spectrum. The signals related to this type of silicon alkoxides usually appear at the lowest chemical shifts between −80 and −130 ppm and these signals are commonly denoted as Q signals. On the other hand, MAPTS has three hydrolysable groups and one functional group, therefore, a maximum of four different signals representing the different products of the hydrolysis and condensation reactions. In this case, signals related to the hydrolysis and condensation of MAPTS are expected in the range between – 40 and – 70 ppm and they are commonly denoted as T signals [36,37].

**Fig. 1:**
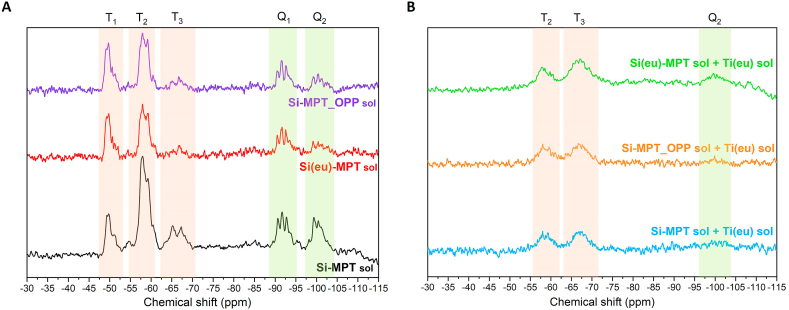
NMR spectra of silicon moieties of sol formulations (A); silicon-titanium sol formulations (B). (For interpretation of the references to colour in this figure legend, the reader is referred to the Web version of this article.)

The liquid ^29^Si NMR spectra of Fig. 1 A shows the effect of the addition of eugenol and OPP (respectively) to the Si-MPT control sol. The main difference observed between the 3 spectra is that when eugenol or OPP are added to the matrix, the condensation of the silicon from the MAPTS is reduced, as the T_3_ signal appearing around −65 ppm decreases in both cases with respect to the one of Si-MPT. Similarly, condensation of TEOS resulted in the decrease of the Q_2_ signal upon addition of OPP and/or eugenol to the Si-MPT sol. On the other hand, Fig. 1 B shows the liquid ^29^Si NMR spectra of formulations containing the titanium moiety. In this case, the intensity of the signal decreases due to the lower concentration of ^29^Si in the formulations [37]. In addition, the three spectra showed no signal for T_1_ and an increment in the T_3_ signal in comparison to the silicon sol before addition of the titanium sol. This suggests that addition of the titanium alkoxide to the formulation promotes hydrolysis and condensation of the MAPTS, independently of the presence of eugenol and OPP, since in the three cases T_3_ signals are present. Regarding condensation of TEOS in the Si–Ti sols, the Q signals get broader, and their intensity significantly decrease, in comparison with Si-MPT sol. This reduction in intensity is so pronounced that the Q signals are close to disappear from the spectra. This could be attributed to the formation of Si–O–Ti bonding that affects the resolution of the spectra broadening the peaks due to a greater distribution of the silicon environments [37]. Furthermore, the only signal that is barely appreciated is the Q_2_ and it seems to be more intense in the formulation containing the highest concentration of eugenol.

Overall, this analysis suggests that eugenol and OPP affect the hydrolysis and condensation reactions that take place in the silicon network when the titanium sol is not included in the formulation. On the contrary, when titanium is added, its effect is more pronounced than that of the addition of either eugenol or OPP.

**Fig. 2:**
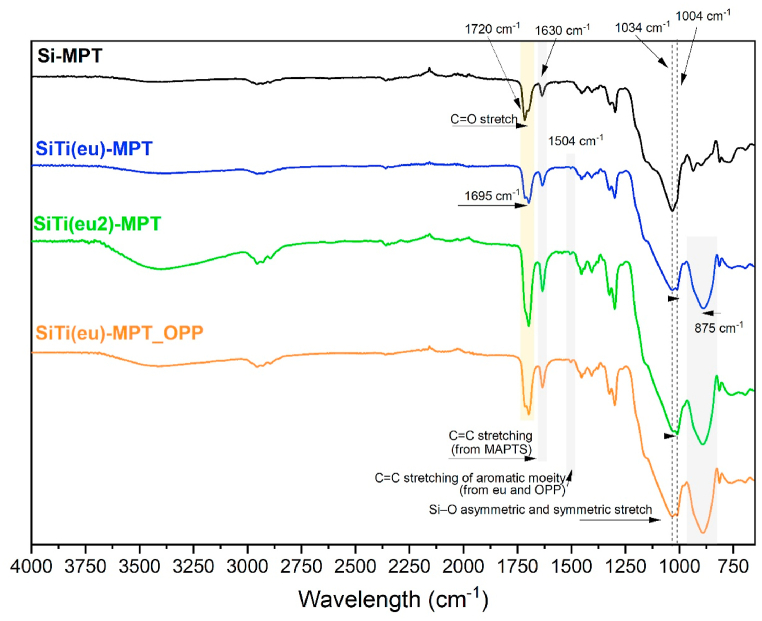
ATR-FTIR of the four coatings under study.

Fig. 2 presents the ATR-FTIR analysis conducted on the four coatings under study. The most notable disparities are observed between Si-MPT and the titanium-containing coatings. A small peak at 1504 cm^−1^ is detected, corresponding to the C=C stretching vibration of the aromatic component (eugenol or OPP) exclusively present in coatings SiTi(eu)-MPT, SiTi(eu)-MPT_OPP, and SiTi(eu2)-MPT. Although this peak is relatively small, it is slightly larger in coatings SiTi(eu)-MPT_OPP and SiTi(eu2)-MPT compared to SiTi(eu)-MPT, indicating lower levels of aromatics in the latter.

Further contrasting Si-MPT with the titanium-containing coatings, a strong band emerges at approximately 875 cm^−1^, associated with the vibrations of Ti–OH and Ti–O–Si. This band is absent in the Si-MPT coating since it lacks titanium. Another distinguishing feature is the broadening and slight shift of the band around 1080 cm^−1^ to 1000 cm^−1^ observed solely in the titanium-containing formulations. This spectral change implies a decrease in the concentration of Si–OH groups and an increased proportion of Si–O–Si bonds. The catalytic influence of titanium is believed to accelerate the sol-gel reaction, leading to the consumption of hydroxyl groups during the condensation process and facilitating the formation of additional Si–O–Si bonds [38].

Moreover, the shift of the carbonyl peak to a lower wavenumber (from 1720 cm^−1^ to 1695 cm^−1^) may indicate a weakening of the bond strength or alterations in the electronic environment surrounding the carbonyl group. This shift suggests the possibility of hydrogen bonding or interactions between eugenol and functional groups within the sol-gel coating.

Measuring the time evolution of a formulation's viscosity is essential for studying the stability of the sol and its pot-life. In addition, it provides knowledge on its susceptibility to gel and its long-term stability to be stored before being deposited on a surface [39]. Moreover, the viscosity of the sol is an important factor to consider when preparing hybrid coatings by the sol-gel method, as it is interrelated with the thickness of the coatings deposited by different type of methods, such as spraying, spin or dip-coating. Some of the benefits of using the dip-coating technique are that it needs a simple design, low-cost equipment, and maintenance. In addition, the films obtained with this method are highly uniform [40].

Dip-coating involves the immersion of a substrate in a solution and its posterior vertical withdrawal [41]. This process consists of five different stages: immersion, dwelling, withdrawal, drying and curing. Usually, the immersion and dwelling steps are not imperative in the process, however the withdrawal speed is a crucial factor as it controls the thicknesses of the coatings. The drying and curing steps are also fundamental aspects to consider because the final properties of the coating may be disturbed if the coating has not achieved the desirable crosslinking [40]. The final thickness of the film is governed by interaction between the entraining forces, draining forces, and the drying of the coating. There are three different regimes describing the formation of a homogenous coating on a surface: viscous flow, drainage, and capillary regimes. The main factors controlling these regimes are the fluid viscosity and the withdrawal speed. In the viscous and drainage regimes the coating is mainly dominated by viscous forces, gravitational attraction, and the surface tension [39,42]. In these regimes, higher withdrawal rates imply higher coating thicknesses. On the other hand, in the capillary region, the coating thickness increases at lower withdrawal speeds [43].

**Fig. 3:**
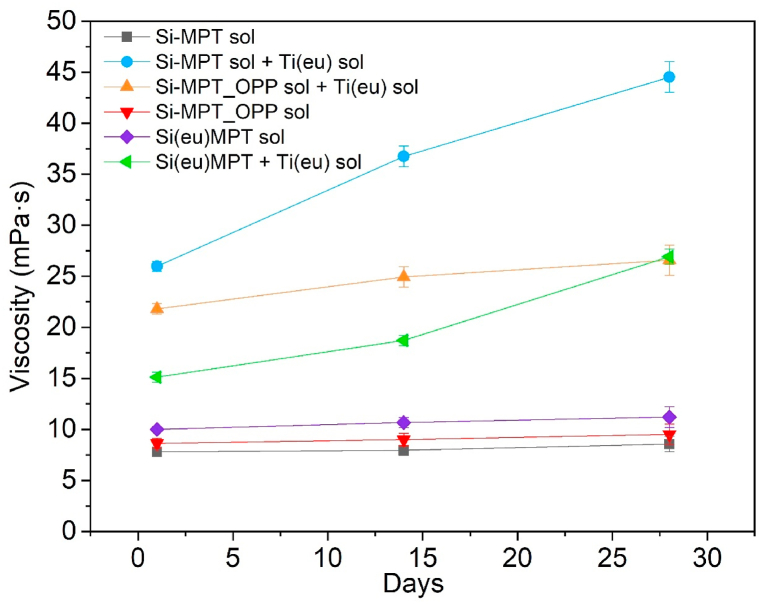
Plot of the viscosity over time of sol formulations under study.

Fig. 3 shows the viscosity of the six sol's formulations studied over time. Si-MPT sol, Si(eu)MPT sol and Si-MPT_OPP sol, show low viscosity and good stability over time. The viscosity increases slightly when eugenol or OPP are added. On the other hand, the mixture Si-MPT sol and Ti(eu) sol, and the mixture Si-MPT_OPP sol and Ti(eu) sol show higher viscosities at initial times. Furthermore, the viscosity of the mixture Si-MPT sol and Ti(eu) sol is almost doubled after 28 days showing the lowest stability over time. In contrast, the viscosity of the mixture Si-MPT_OPP sol and Ti(eu) sol even though it is similar to the one of the mixture Si-MPT sol and Ti(eu) sol at initial times, it remains more stable over time. The only difference among the two mixtures is the addition (or not) of the OPP. The addition of OPP significantly decreases the formulation's viscosity. Therefore, the data suggest that OPP is acting as a reactive diluent, a substance usually included in formulations to lower viscosity avoiding the use of flammable solvents. In addition to lower viscosity, reactive diluents also need to be compatible with the formulation, have low volatility, and the ability to participate in the curing process among others [44]. The reactive diluents can be classified into two categories: monofunctional and polyfunctional reactive diluents. Typically, the monofunctional reactive diluents demonstrate a decrease in modulus and an enhanced ductility. In contrast, di- and multi-functional reactive diluents yield the opposite outcome [44]. OPP is a monofunctional molecule that has a free –OH group that is able to condensate with the -Si-OH free groups upon curing. Thus, it is expected that the coating containing this molecule will present a higher ductility. Finally, the combination of Si(eu)MPT sol and Ti(eu) sol, shows an initial viscosity lower than the combination Si-MPT sol and Ti(eu) sol, and even lower than Si-MPT_OPP sol and Ti(eu) sol mixture.

The viscosity results show that OPP is stabilizing the formulations viscosity and making the formulation to have a longer pot-life. In addition, higher concentrations of eugenol (SiTi(eu)-MPT vs SiTi(eu2)-MPT, Table 1, Table 2) in the formulations containing titanium are also thinner and more stable over time.

With respect to the thickness (Table 3), the thickest coating is SiTi(eu)-MPT. SiTi(eu)-MPT_OPP gives a thickness slightly lower as it contains a reactive diluent in its formulation that upon curing reacts with the free Si–OH groups helping to obtain a denser and a higher crosslinked network. Si-MPT and SiTi(eu2)-MPT have a low thickness which remains in the middle of the other two coatings.

**Table 3:** Thickness of the different coatings under study after deposition by dip-coating at a withdrawal speed of 5 cm/min.

Coatings	Thickness (μm)
**Si-MPT**	1.80 ± 0.11
**SiTi(eu)-MPT**	2.05 ± 0.06
**SiTi(eu)-MPT_OPP**	1.07 ± 0.23
**SiTi(eu2)-MPT**	1.78 ± 0.10

The contact angle was evaluated to understand if there was any difference in the wettability of the different selected coatings. SiTi(eu2)-MPT was excluded from this analysis due to the antibacterial results (see below). Coating Si-MPT showed a larger contact angle, 71.7° (standard deviation of 0.7), i.e., was more hydrophobic, than coatings SiTi(eu)-MPT and SiTi(eu)-MPT_OPP (66.9 ± 1.4° and 65.2 ± 1.2° respectively) (Table 4). This might be related to a larger presence of hydroxyl groups at the surface in the latter, as both eugenol and OPP molecules have originally hydroxyl groups.

**Table 4:** Water contact angle (WCA) and mechanical properties by nanoindentation (average and standard deviation).

Coating	WCA [°]	HIT [MPa]	E* [GPa]	EIT [GPa]	h_max_ [nm]	LC3 [N]
Glass slide	66.8 ± 4.5	–	–	–	–	–
Si-MPT	71.7 ± 0.7	350 ± 63	1.82 ± 0.14	1.65 ± 0.13	194 ± 14	–
SiTi(eu)-MPT	66.9 ± 1.4	279 ± 12	3.70 ± 0.19	3.37 ± 0.18	171 ± 9	0.35 ± 0.05
SiTi(eu)-MPT_OPP	65.2 ± 1.2	293 ± 23	4.12 ± 0.28	3.75 ± 0.25	163 ± 9	0.50 ± 0.09

NOTE: HIT Hardness; E* Plane strain modulus; EIT Elastic Indentation Modulus; h_max_ maximum indentation depth; LC3 Critical Load.

**Fig. 4:**
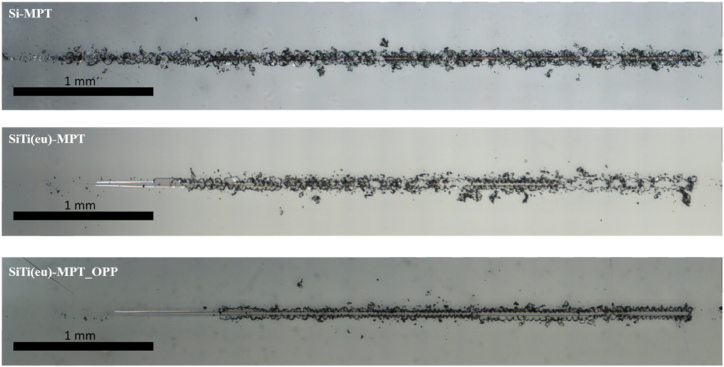
Scratch test tracks in coating Si-MPT (above), SiTi(eu)-MPT (middle) and SiTi(eu)-MPT_OPP (below).

In terms of hardness, coating Si-MPT also tended to show a larger hardness value, 350 ± 63 MPa, than both coating SiTi(eu)-MPT_OPP, 293 ± 23 MPa, and SiTi(eu)-MPT, 279 ± 12 MPa, although differences were not significant (Table 4). On the other hand, coating Si-MPT was remarkably stiffer, as measured in the Young Modulus, than the other two coating variations. This can also be observed in the scratch test tracks (Fig. 4), where Si-MPT shows quite a fragile break up, while the spalling out of the SiTi(eu)-MPT and specially SiTi(eu)-MPT_OPP coatings, which shows a better adhesion, is more gradual.

In addition, when comparing coatings SiTi(eu)-MPT and SiTi(eu)-MPT_OPP, as it was mentioned in the viscosity section, with the addition of monofunctional reactive diluents such as OPP, a coating with higher modulus was expected. This is now here confirmed since the Young Modulus for SiTi(eu)-MPT is 3.70 ± 0.19 GPa while for SiTi(eu)-MPT_OPP is 4.12 ± 0.28 GPa.

### 3.2 Biological properties of the coatings

The antibacterial activity was assessed for coatings Si-MPT, SiTi(eu)-MPT, and SiTi(eu)-MPT_OPP against *Escherichia coli* and *Staphylococcus aureus*, Gram-negative and Gram-positive bacteria, respectively.

All the three surfaces showed a strong antibacterial activity with reduction of the number of *E. coli* bacteria above 99.99 % after 24 h in all cases. The antibacterial activity was highest for coating SiTi(eu)-MPT (99.9998 % reduction), though close to that of Si-MPT and SiTi(eu)-MPT_OPP, with a 99.9965 % and 99.9890 % reduction, respectively.

**Fig. 5:**
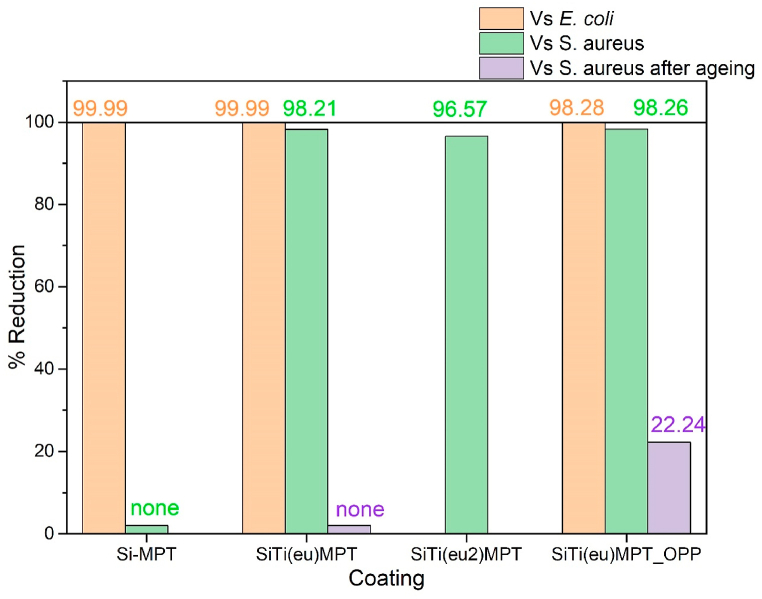
Bacterial reduction percentages on the coatings of *E. coli*, *S. aureus* and *S. aureus* after aging (durability tests).

On the other hand, the Si-MPT coating did not show any antibacterial activity against the Gram-positive *S. aureus*, unlike coatings SiTi(eu)-MPT, SiTi(eu2)-MPT and SiTi(eu)-MPT_OPP, with reductions of 98,21 %, 96,57% and 98,26 % respectively (Fig. 5).

The first remarkable result to note is the very different performance of coating Si-MPT, which showed strong antibacterial activity against *E. coli*, but none against *S. aureus*. A different antibacterial performance against *E. coli* and *S. aureus* has been previously described, e.g., against different plants extracts [45]. Typically, the antibacterial effect is associated to actions at the level of the bacterial membrane, a crucial structure for cell survival. The structure and molecular components of the bacteria membrane and cell wall differ between Gram-positive and Gram-negative bacteria [46]. Due to their distinctive structure, Gram-negative bacteria are more resistant than Gram-positive bacteria, and cause significant morbidity and mortality worldwide. The majority of the World Health Organization list of antibiotic-resistant priority pathogens is Gram-negative [47].

It is known that phenolic compounds, generally show antimicrobial activity against Gram-positive bacteria [48]. Phenolic groups are present in eugenol and OPP, but not in coating Si-MPT, which could explain the lack of antibacterial activity observed for Si-MPT against *S. aureus*.

Furthermore, it has been reported that in the case of compounds like eugenol, its antimicrobial activity is also linked to their free hydroxyl groups and to the presence of a double bond in the α,β positions of the side chain and to a methyl group located in the γ position [23,49,50]. This could explain the additional activity found in SiTi(eu)-MPT against *E. coli* as compared with the other two coatings. However, coating Si-MPT have also shown a significant reduction of *E. coli*, albeit to a lesser extent, which could be linked to the presence of the methacrylate groups. It has also been reported elsewhere that eugenol exhibit higher activity against Gram-negative bacteria than Gram-positive bacteria [51], further validating the results from this study.

Coating SiTi(eu)-MPT, which contains a lower amount of eugenol showed a similar or slightly better performance than SiTi(eu2)-MPT. Thus, since the presence of additional eugenol did not result in improved properties, the coating SiTi(eu2)-MPT was not further characterized (e.g., mechanical properties). In relation to coating SiTi(eu)MPT_OPP, it has shown a performance close to that of SiTi(eu)-MPT, slightly more effective against *S. aureus*, but less against *E. coli*. The former might be linked to the mix and surface density of phenolic compounds in both eugenol and OPP, while the latter might be linked to different mix and density of methyl and hydroxyl functional groups on the surface.

Additionally, all three coatings were found to be non-cytotoxic according to ISO 10993-5:2009 [34].

Concerning the influence of the surface wettability, surfaces of coatings SiTi(eu)-MPT and SiTi(eu)MPT_OPP were a little more hydrophylic than the Si-MPT surface (Table 4). However, differences were almost within the error margin of the measuring equipment (2-3°). Besides the bactericidal test methodology comprises placing a cover on top of the bacteria covered test samples, thus it can be concluded that the effect of the surface wettability on the bacteria adhesion was minimal in this study.

**Fig. 6:**
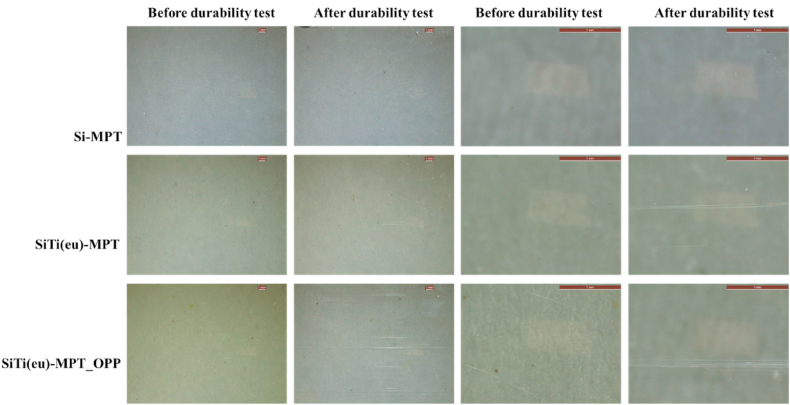
Coating surfaces at the optical microscopy before and after the tribology (durability) tests. NOTE: The scale bar corresponds to 1 mm.

Next, we tested durability of the activity of the different coatings, an important parameter that is often overlooked in the scientific literature. For that, the antimicrobial activity against *S. aureus* was tested after the tribology tests in cleaning media and compare to that measured prior to the treatment. Coatings SiTi(eu)-MPT and SiTi(eu)-MPT_OPP exhibited unsatisfactory aging behaviour. Specifically, coating SiTi(eu)-MPT showed no antibacterial activity, while SiTi(eu)-MPT_OPP's activity was diminished to a reduction of 22 % of bacteria growth. Additionally, it could be observed that the coatings presented surface scratches, along the longitudinal axis of the tribology tests (Fig. 6). This was reflected in the surface roughness which raised from Ra 3.3 to Ra 27.5 μm in coating SiTi(eu)-MPT_OPP, much more than in coating SiTi(eu)-MPT, from Ra 2.1 to Ra 3.3 μm. This might be caused by the release of some hard particles from the coatings, their entrapment between the coating and the reciprocating sponge, acting as third body abrasive particles.

**Fig. 7:**
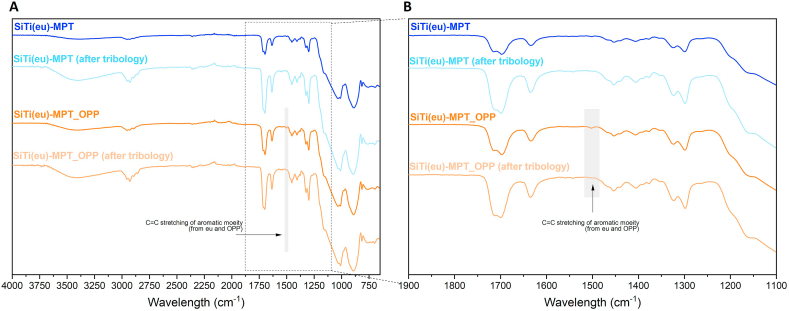
ATR-FTIR of the coatings SiTi(eu)-MPT and SiTi(eu)-MPT-OPP before and after the aging experiment was performed. Spectra between 4000 and 650 cm ^−1^ (A) and spectra between 1900 and 1100 cm ^−1^ (B).

Fig. 7 A depicts a comparison between the ATR-FTIR spectra of coatings SiTi(eu)-MPT and SiTi(eu)-MPT_OPP before and after undergoing the aging test. Minimal differences are seen between the spectra, except for a potential change in the broad band around 3500 cm^−1^, which appears to increase following the durability test. This change is believed to be a result of the coating absorbing moisture from the liquid used in the test. Despite the coatings showing signs of damage and reduced antimicrobial effectiveness after the durability test, there are minimal observable alterations in the structure of the coatings. The only notable change is the absence of a small peak at 1504 cm^−1^, which corresponds to the C=C asymmetric stretch of aromatic compounds like eugenol/OPP [52] (Fig. 7 B). These compounds are primarily responsible for the antimicrobial properties of the coatings.

**Fig. 8:**
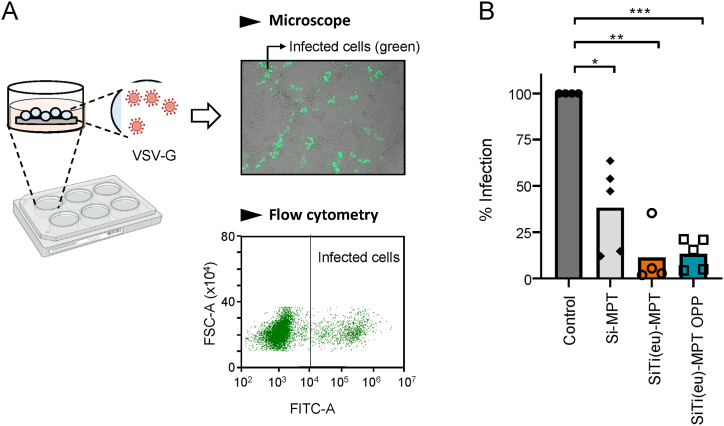
In vitro antiviral activity of the coating agents. A) Experimental set up to assess the capacity of the coatings to prevent viral infection of mammalian cells. Cell infection leads to expression of the green fluorescent protein (GFP). Infected cells (shown in green in the microscope image, top right) are quantified by flow cytometry (bottom right). B) Percentage of infected cells upon incubation of the PsVs with a surface coated with different coatings shown in the plot. Control sample refers to the untreated surface. The bars represent mean values of at least four independent measurements. ****<0.0001, ***<0.0002, **<0.0021 y *<0.0332 indicates significance compared to control sample by ANOVA. (For interpretation of the references to colour in this figure legend, the reader is referred to the Web version of this article.)

Finally, in order to test the virucide activity of the coating, we first adapted a cell-entry inhibition assay that includes a preincubation step of the pseudovirus (PsVs) with the different coating agents. We used pseudotyped Vesicular Stomatitis Virus G (VSV-G), an enveloped virus capable of infecting a wide variety of mammalian cells. Several drops containing VSV-G PsVs were placed on glass cover surfaces and incubated for 1 h at 37 °C to allow PsV attachment. The excess of PsV was gently removed and the capacity of the bound PsVs to infect HeLa cells monitored by confocal microscopy and quantified using flow cytometry (Fig. 8A). Consistent with a virucidal effect, coating of the glass covers led to significant decreases in the number of infected cells (Fig. 8B). Specifically, we observed an average of 63 %, 90 % and 87 % decrease in the number of infected cells upon coating with Si-MPT, SiT(eu)-MPT and SiTi(eu)-MPT_OPP, respectively, highlighting the capacity of these coating agents to interfere with cell infection.

## 4 Conclusions

This study demonstrates that both biocide agents eugenol and OPP can be successfully incorporated into sol-gel silicon-titanium- methacrylate functional coating compounds on both glass and stainless-steel substrates.

The incorporation of these compounds did not interfere in the hydrolysis and condensation reactions of the sol, and the resulting crosslinked coating network as confirmed by liquid ^29^Si NMR and ATR-FTIR. Additionally, these biocide agents were not trapped in the coating network but kept their functionality at the outer surface. Thus, the initial hypothesis that upon its incorporation into the matrix, eugenol may still act as a killing and biocide agent has been confirmed by the results.

Selected coatings combining eugenol and OPP further proved to cause very significant reductions of *E. coli* (Gram-negative) and *S. aureus* (Gram-positive) bacteria concentrations at the surfaces, while being non-cytotoxic. It was also found that the base coating, Si-MPT, present a remarkable antibacterial activity against *E. coli*, but none against *S. aureus*. What is more and equally important, results in this study demonstrate the capacity of SiTi(eu)-MPT and SiTi(eu)-MPT_OPP coating to interfere with cell viral infection. However, the endurance of the antimicrobial activity in daily operational conditions, an often-overlooked feature, has proved to be a challenge that should be addressed in future research.

In conclusion, our results support the suitability of working with natural wide spectrum antimicrobial agents, like eugenol, as well as the importance of considering already from early stages of the design phase the endurance along the operative life of the coatings.

## Data availability statement

Data will be made available on request.

## CRediT authorship contribution statement

**Ana Suárez-Vega:** Conceptualization, Data curation, Formal analysis, Investigation, Methodology, Validation, Writing – original draft, Writing – review & editing. **Gemma Berriozabal:** Investigation, Methodology, Resources, Writing – review & editing. **Juan Perez de Iriarte:** Formal analysis, Investigation, Methodology, Validation. **Jaione Lorenzo:** Investigation, Methodology, Resources. **Noelia Álvarez:** Conceptualization, Data curation, Investigation, Methodology, Resources, Validation, Writing – original draft, Writing – review & editing. **Santiago Dominguez-Meister:** Conceptualization, Formal analysis, Investigation, Methodology, Resources, Supervision, Writing – review & editing. **Sara Insausti:** Data curation, Investigation, Methodology. **Edurne Rujas:** Conceptualization, Data curation, Validation, Writing – original draft, Writing – review & editing, Investigation, Methodology, Resources. **Jose L. Nieva:** Conceptualization, Funding acquisition, Investigation, Supervision, Methodology. **Marta Brizuela:** Conceptualization, Funding acquisition, Investigation, Methodology, Visualization. **Iñigo Braceras:** Conceptualization, Formal analysis, Funding acquisition, Investigation, Methodology, Project administration, Supervision, Validation, Writing – original draft, Writing – review & editing.

## Declaration of competing interest

The authors declare that they have no known competing financial interests or personal relationships that could have appeared to influence the work reported in this paper.
